# The Effect of Septoplasty and Turbinoplasty on Pulmonary Function Test– A Hospital-Based Interventional Study

**DOI:** 10.22038/ijorl.2025.83789.3825

**Published:** 2025

**Authors:** Suvamoy Chakraborty, Nayana Sarma, Sauradeep Das, Vijay N Nongpiur, Manu C Balakrishnan, Zareen Lynrah, Abhijeet Bhatia

**Affiliations:** 1 *Department of Otorhinolaryngology, North Eastern Indira Gandhi Regional Institute of Health and Medical Sciences, Shillong-793018,* * Meghalaya, India.*; 2 *Department of T.B and Respiratory Medicine, North Eastern Indira Gandhi Regional Institute of Health and Medical Sciences, Shillong-793018, Meghalaya, India.*

**Keywords:** Septoplasty, Pulmonary Function Test, Sino-bronchial reflex, Deviated nasal septum, Nose

## Abstract

**Introduction:**

Deviated nasal septum (DNS) is common in the population and at times can warrant a need for septal surgeries. It has been hypothesised that DNS increases the post-nasal discharge, leading to increased sino-bronchial reflexes. This leads to lower respiratory tract inflammation and infections. The current study has been done to confirm the above hypothesis and to evaluate the improvement after septal surgeries among the patients.

**Materials and Methods:**

72 patients, above 18 years of age, who had undergone a septal correction surgery were included in our study. Pulmonary function tests (PFT) like FVC, FEV1, FEV1/FVC, FEF25%-75%, and PEF were used to evaluate the patients pre-operatively, 1 month post-operatively, and 2 months post-operatively. Additionally, the Nasal Obstruction Evaluation Scale (NOSE) was used to assess the improvement in PFT, comparing the pre-operative and 2 months post-operative PFT. The study was conducted from November 2022 to May 2023. All data were recorded and analysed using SPSS version 21.0.

**Results:**

All PFT indices showed improvement on both the 1^st^ month (p>0.05) and 2^nd^ month (p<0.05) post-operatively. All patients had an improvement in the NOSE score 2 months post-operatively (p<0.001). Among all the patients, only the overweight and obese patients had a lower degree of improvement in PFT.

**Conclusion:**

Our study thus concludes that septal surgeries have a positive impact on the Lower Respiratory Tract, thus confirming our hypothesis.

## Introduction

A septal deviation of clinical significance is defined as a deviation that amounts to severe obstruction, which is not resolved by medical therapy (1). The prevalence of septal deviations in the literature ranges between 26% and 97% due to varying morphological features and the extent of deviation (2).

Deviated nasal septum (DNS) and its pathological sequelae, such as hypertrophied inferior turbinate, lead to alteration of the respiratory mechanics and changes in the composition of arterial blood (3,4). DNS leads to pathological sino-bronchial reflex and post-nasal discharge, which irritates the lower airways. Unfiltered and cold air due to mouth breathing because of obstruction of the nose causes airway irritability (5). These cause changes in standard pulmonary functions, showing alterations in the Pulmonary Function Test (PFT) (6). Impairment in drainage of nasal secretions due to DNS leads to recurrent respiratory infections with relative hypoxia and hypercapnia, generating inflammatory cytokines, free nitrogen, and oxygen radicals, which can lead to impaired pulmonary functions (4). Various studies have revealed varied results on the change in pulmonary function test post relief of upper airway obstruction. Septoplasty refers to a surgical manipulation and/or removal of deviated cartilage and/or bone forming the septum to correct a nasal septal deformity (7). Various pieces of literature have shown alteration or improvement in PFT post-upper airway obstruction relief. The PFT were done during a period of 1 month to 6 months in the studies reviewed. Pre-operative and post-operative forced vital capacity (FVC), forced expiratory volume in 1 second (FEV1), ratio of FEV1/FVC, peak expiratory flow (PEF), forced expiratory flow at 50% of FVC (FEF50%), forced inspiratory flow at 50% of FVC (FIF50%), and the ratio of maximum expiratory to inspiratory flow at 50% of FVC (FEF50%/FIF50%) were compared in different studies. It has also been seen in studies that post-septoplasty, there is improvement in sleep disorders as noted in polysomnographic studies (1,3,5,8). In our study, we have compared the improvement with a validated quality of life questionnaire and a Pulmonary functional test to quantify both subjective and objective improvements of these patients. *Aims and objectives:*

- To know the improvement in the lower airway function of patients undergoing septoplasty by using the pulmonary function test.
- To see the correction in deviation of the nasal septum after septoplasty and consequent symptoms.

## Materials and Methods

### Methodology:

Study design- A hospital-based pre-post interventional study among the septoplasty patients.

The study group included all routine patients who were willing to participate in the study. *Inclusion criteria: *1) Patients with a deviated nasal septum willing to undergo surgery. 2) Patients above 18 years of age.

*Exclusion criteria*:1) Patients with lung diseases/ disorders. 2) Patients with associated paranasal sinus disorders. 3) Patients with a mass in the nasal cavity.

### Study procedure:

A detailed clinical history and Ear, Nose, Throat (ENT) clinical examination were performed. Patients with DNS were taken for Diagnostic nasal endoscopy (DNE) using a Storz 0-degree endoscope (Karl Storz SE & Co., Germany). DNS was graded as: Grade 1- DNS not reaching the lateral nasal wall, Grade 2- DNS touching the lateral nasal wall and inferior turbinate, and Grade 3- DNS compressing the inferior turbinate.(9) Patients fulfilling the inclusion criteria were counselled for participation in the study; all patients counselled gave consent for the same and were prepared for the surgery. PFT was done using a TrueFlow Spirometer (NDD Medical Technologies, USA) to determine the pre-operative status of the lung function. NOSE score, which is a commonly used quality of life (QOL) questionnaire, was used for all the participants in the study.(10) This is an easy-to-use questionnaire validated for septoplasty and septo-rhinoplasty patients. Each item is scored from 0 to 4, where 0 indicates not a problem and 4 indicates a severe problem. The scale was then scored from 0 to 100 by multiplying the raw score by 5 (11,12). Patients were taken up for surgery (septoplasty) under local or general anaesthesia. Following surgery, bilateral nasal cavities were packed with Merocel, which were subsequently removed on post-operative day 2; and during those 2 days, patients were kept on intravenous antibiotics. After pack removal, oral antibiotics were prescribed for seven days. Patients also received normal saline washes twice daily after pack removal, and the procedure for wash was taught to the patient and the caretaker. Patients were discharged on the 3^rd^ or 4^th^ post-operative day and were asked to review in the ENT OPD after 1 month for clinical evaluation. PFT was also repeated to know the post-operative status of lung function at one month. Patients were followed up again at the end of two months of surgery for clinical evaluation based on symptoms, clinical findings, DNE, NOSE score, and PFT.

 An ENT Proforma with history and clinical examination was used for the patients, along with another questionnaire, which included the Nasal Obstruction Evaluation Scale (NOSE) score items and PFT indices. The proforma and questionnaire were in English. Questionnaires were given to the patients to fill up. For patients having a language barrier, the questionnaires were filled out by the investigator after conversing in the local language. The study was conducted over one and a half years, starting from November 2022 to May 2023. 

Ethical clearance for the study was obtained from the Institution Ethics Committee (NEIGR/IEC/M7/T5/2022), and the study was done following the Helsinki protocol.

*Sample size:* Sample size was calculated by using online open EPI Software (https://www.oprnepi.com/SampleSize/SSPropor.htm). From a study done by K. A. Arifa et al.,(1) the pre-operative and post-operative means of FEV1/FVC and standard deviations were taken, and the sample size was calculated. With a two-sided confidence interval of 95% and power of 80%, a sample size of 72 patients was achieved. Consecutive sampling was done among the patients who visited our Outpatient Department, fulfilling the inclusion criteria. 

*Statistical analysis*: All the data were entered in Microsoft Excel, and the analysis was performed on SPSS software version 21. Descriptive statistics were reported for demographic variables such as age, sex, ethnicity, and BMI, with continuous variables presented as mean ± standard deviation (SD), and categorical variables as frequencies and percentages. A P<0.05 indicated statistical significance. The McNemar test was done to assess the changes in paired categorical data (symptoms) between pre-operative and post-operative data. Additionally, after using the Shapiro-Wilk test to confirm the normality, paired t-tests were done to compare the mean of PFT indices (FVC, FEV1, FEV1/FVC, FEF25%-75%, PEF) and the NOSE score values between different time points (Pre-operative vs. 1-month post-operative, Pre-operative vs. 2-month post-operative, and 1-month post-operative vs. 2-month post-operative) to determine if the changes in the PFT indices over time are statistically significant. A p-value < 0.05 indicated statistical significance. 

## Results

**Fig 1:**
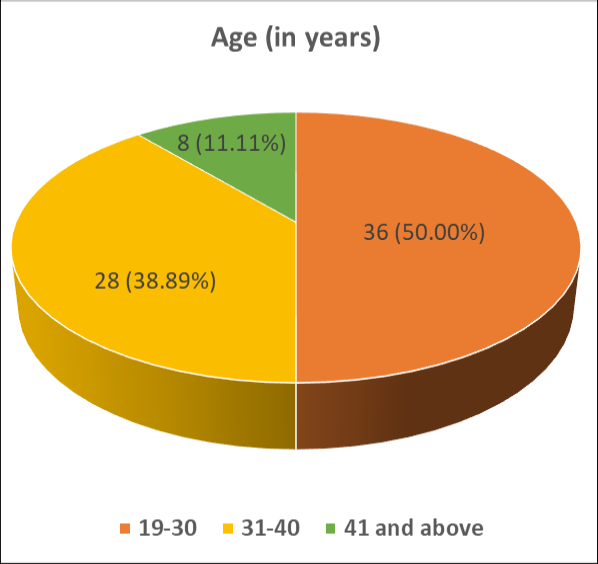
Frequency distribution according to age groups

A total of 72 patients were included in the study, with age range of 19 years to 52 years, with a mean age of 30.58 ± 7.55 years. For analysis, patients were divided into three age groups as shown in Figure 1. 19 (26.39%) patients were female, and 53 (73.61%) patients were male. As PFT values are affected by the body mass index (BMI), patients were also divided into four groups based on BMI, with 10 patients in the underweight category, 40 patients in the normal range, 20 patients in the overweight category, with 2 patients in the obese category.

**Fig 2:**
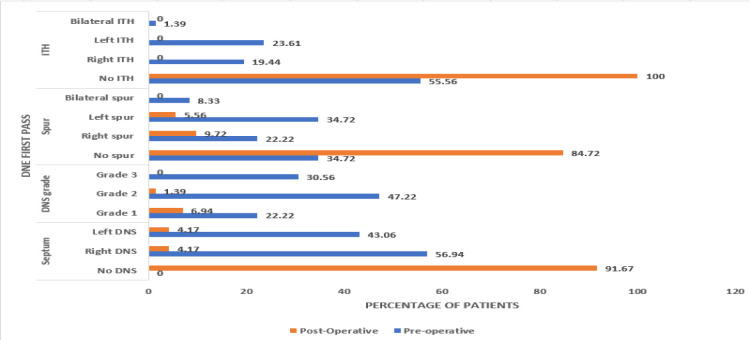
Particulars of 1st pass DNE and the frequencies of appearance in the study population; pre-operative and 2 months post-operative.

NOSE score was calculated for each patient, pre-operatively and 2 months post-operatively. The mean NOSE score pre-operatively was 62.36 ± 2.63, and 2 months post-operatively was 11 ± 1.19, with a significant improvement with a p-value of <0.001. Improvement of DNS, spur, and Inferior turbinate hypertrophy (ITH) were present post-operatively as shown in Figure 2, with a significant p-value of <0.001. All 72 patients underwent septoplasty. Along with septoplasty, ITH was reduced surgically by inferior turbinoplasty or submucosal diathermy for 9 (12.5%) patients. 70 patients were operated under local anaesthesia, while 2 patients were operated under general anaesthesia. 

Comparison of the mean of PFT indices revealed improvement at both the 1^st^ and 2^nd^ months post-operatively when compared to preoperative PFT values; however, improvement in the 1^st^ month was not statistically significant. Mean values of PFT indices and their statistical analysis are represented in Table 1.

**Table 1:** Comparison of the mean of PFT indices pre-operatively and 1-month and 2 months post-operatively. N=72

**Sl. No.**	**PFT particulars**	**Preoperative**	**1 month postoperative**	**p-value**	**2 months postoperative**	**p-value**
1.	FVC (L)	3.47±0.78	3.47±0.75	0.95	3.74±0.79	0.03*
2.	FEV1 (L)	2.99±0.71	3.02±0.68	0.81	3.30±0.70	<0.001*
3.	FEV1/FVC (%)	0.85±0.66	0.86±0.63	0.59	0.88±0.57	0.01*
4.	FEF25%-75% (L/s)	3.71±1.41	3.74±1.36	0.91	4.24±1.39	0.02*
5.	PEF (L/s)	8.02±2.25	8.47±2.49	0.24	9.24±2.26	<0.001*

Test used: Paired t-test. *- significant p-value

PFT indices were compared between preoperative and 2-month postoperative based on various age groups, genders, grades of DNS, and BMI, as shown in Table 2. Absolute values of PFT indices post-operatively were noted to be lower for females as compared to males; however, improvement of PFT indices post-operatively was significant for both genders (p = < 0.001 for both genders). Significant improvement of PFT indices was noted in various categories of patients, except for patients ≥ 41 years (FVC p =0.17, FEV1 p = 0.8, FEF 25%-75% p =0.27) and obese patients (FVC p =0.45, FEV1 p = 0.2, FEF 25%-75% p =0.41, PEF p=0.27), in whom improvement in FVC, FEV1, and FEF 25%-75% was not significant (PEF was not significant in obese patients). It was observed that FEV1 was also insignificant in overweight patients (p = 0.48). Improvement in FEV1/FVC was significant in all categories of patients (p = <0.001)

**Table 2:** Comparison of Pre-operative and 2 months post-operative values of FVC, FEV1, FEV1/FVC, FEF 25%-50%, and PEF on various categories of patients. N=72

**Sl.** **No.**	**Characteristics**	**Category (n)**	**p- value** **FVC**	**p- value** **FEV1**	**p- value** **FEV1/FVC**	**p- value** **FEF 25%-50%**	**p- value** **PEF**
**1.**	Age (years)	19-30 (36)	<0.001	<0.001	<0.001	<0.001	<0.001
		31-40 (28)	<0.001	<0.001	<0.001	<0.001	<0.001
		≥41 (8)	0.17*	0.80*	<0.001	0.27*	<0.001
**2.**	Gender	Female (19)	<0.001	<0.001	<0.001	<0.001	<0.001
		Male (53)	<0.001	<0.001	<0.001	<0.001	<0.001
**3.**	Pre-operative grade of DNS	Grade 1 (16)	<0.001	<0.001	<0.001	<0.001	<0.001
		Grade 2 (34)	<0.001	<0.001	<0.001	<0.001	<0.001
		Grade 3 (22)	<0.001	<0.001	<0.001	<0.001	<0.001
**4.**	BMI (kg/m^2^)	Underweight (10)	<0.001	<0.001	<0.001	<0.001	<0.001
		Normal range (40)	<0.001	<0.001	<0.001	<0.001	<0.001
		Overweight (20)	<0.001	0.48*	<0.001	<0.001	<0.001
Obese (2)	0.45*	0.20*	<0.001	0.41*	0.27*

Test used: Paired t-test. *- p-value not significant

## Discussion

The deviated nasal septum is one of the most commonly diagnosed disorders in the ENT clinics, which may or may not be associated with external nasal deformity (9,13). A significant DNS alters the normal physiology of the nose and the paranasal sinuses and also imparts its effect on the pulmonary function of the individual (14,15). The functional evaluation of obstruction of the nose can be done in two ways: either objectively or subjectively (11). Amongst the subjective scores, the NOSE score was used for the patients in our study. On comparison, the mean NOSE score pre-operatively is 62.36 ± 2.63, while 2 months post-operatively, it is 11 ± 1.19, with a p-value of <0.001, indicating a significant improvement of the patients symptomatically after septoplasty. Similarly, in a study by Sobh et al., the pre-operative mean NOSE score was 63.2 ± 10.9, and the post-operative mean was 27.0 ± 6.1 with a p-value of <0.001(16). PFT, mostly used to determine lung function, can indirectly point to an obstruction in the nasal cavity. Nasal obstruction can affect pulmonary functions, which can be via a neural or mechanical action (5,16,17). It has been seen that packing of the nasal cavities, which may be done following any surgical intervention in the nose or in the management of epistaxis, can cause hypoxia, which is due to the nasopulmonary reflex (18,19). Multiple studies with PFT at different intervals ranging from 2 weeks to 6 months post-operatively have been published (5,13,16). Different studies revealed different improvement levels in the various indices of PFT (Table 3), while a few studies did not have significant improvement in all particulars (8,20). 

**Table 3:** Comparison of the p-values of various studies and the current study

**Study by**	**FVC**	**FEV1**	**FEV1/FVC**	**PEF**
Elsherif H et al.(2019)(21)	< 0.001	<0.001	<0.001	-
Jarandikar A et al.(2020)(22)	<0.001	<0.001	<0.001	not significant
Tuzuner A et al. (2016)(3)	0.191	0.428	0.441	<0.001
Bulcun E et al. (2010)(5)	0.049	0.007	0.125	0.007
Nanda et al. (2019)(23)	0.025	0.049	0.416	-
Sobh et al.(2021)(16)	<0.001	<0.001	0.7	-
Panicker et al.(2018)(17)	<0.001	<0.001	<0.001	0.005
Present study (1-month post-operative )	0.95	0.81	0.59	0.24
Present study (2 months post-operative)	0.03	<0.001	0.01	<0.001

From the above table, it is evident that lung functions are affected in nasal obstruction, and improvement in the same is noted after correction of the obstruction. Other than septoplasty, similar findings were also noted in cases with chronic rhinosinusitis, where improvement in PFT indices was noted following sinus surgery (21). In our study, a paired t-test was used to find the variations in pre-operative and post-operative PFT, which revealed a significant improvement in almost all indices of PFT at 2-month intervals from surgery, which was not observed at 1 month after surgery, which may be due to post-operative inflammation. Obstruction of the nose causes an increase in the negative pressure in the upper airway and leads to a collapse of the airway during inspiration at the level of the pharynx, which reduces the flow of air to the lower airways. It may cause nighttime hypoxemia or free radical injury in the vasculature and even lead to cardiac, endocrine, and neurological disorders without proper management (6). Various studies have evaluated the effect of nasal obstruction on pulmonary function indices and the impact of the elimination of nasal obstruction on pre-operative values (16).

*Strengths of the study:* This study was a prospective interventional study, and all surgeries were performed by a single team of surgeons. All the pulmonary function tests were done in the same centre by the same team of technicians with the same apparatus.

*Limitations of the study:* Due to the wide geographical distribution of patients, follow-up of patients was difficult and was hence limited to 2 months post-operatively.

## Conclusion

This study reveals an improvement in PFT following septoplasty, indicating an effect on the lower airways by upper airway obstruction. Surgical correction of DNS not only relieves the nasal symptoms but also improves pulmonary function. Larger studies are however, needed to study the impact of further categories like type and extent of surgery on PFT, correlations between NOSE score or other subjective scoring systems with objective tests, and correlation of PFT and modern objective tests like rhinomanometry and acoustic rhinometry.
